# Availability, price, and affordability of WHO priority maternal and child health medicine in public health facilities of Dessie, north-East Ethiopia

**DOI:** 10.1186/s12911-020-01247-2

**Published:** 2020-09-11

**Authors:** Abel Demerew Hailu, Solomon Ahmed Mohammed

**Affiliations:** 1 Department of Pharmacy, Dessie Health Science College, Dessie, Ethiopia; 2 Department of Pharmacy, College of Health Science, Wollo University, Dessie, Ethiopia

**Keywords:** Availability, Affordability, Price, Medicines, Maternal, And child

## Abstract

**Background:**

Access to health care is a fundamental human right, and the provision of affordable, high-quality, and appropriate medicines for maternal and child health is a vital component of a well-functioning health system. The study assessed the availability, price, and affordability of WHO priority maternal and child medicines in public health facilities, Dessie, North-East Ethiopia.

**Methods:**

A retrospective cross-sectional study design was conducted in Dessie town from November 2018 to February 2019. A standard checklist adapted from the Logistics Indicator Assessment Tool and WHO/HAI was used to collecting data on the availability, affordability, and price of 45 priority life-saving medicines from eight public health centers and two public hospitals. Descriptive statistics (percent and median) were computed for availability and prices. Affordability was reported in terms of the daily wage of the lowest-paid unskilled government worker.

**Results:**

Twenty-two medicines were not found at all in public health facilities. The overall availability of WHO priority maternal and child medicines was 34.02%. The mean number of stock-outs was 3.9, and the mean number was 128.9 days. The mean average point availability was 33.5%, and 7 medicines stock out on the days of assessment. From WHO priority maternal and child medicines, 4 (40%) of the products were unaffordable and 5 (55.5%) had higher prices than international prices. Ceftriaxone 1 g, ceftriaxone 500 mg, and hydralazine 20 mg injection required wages of 6.58, 8.01, and 5.02 to cover specific maternal health problems respectively. The median price ratio of priority lifesaving maternal and child medicines in public health facilities ranged from 0.65 to 3.19.

**Conclusions:**

The average mean period and point mean availability was very low. The available products were encountered with a high number of stock-outs and unaffordable. The strict control of inventory is recommended to have a steady supply of these essential medicines and improve the quality of health services.

## Background

Maternal and child health (MCH) focuses on the determinants, mechanisms, and systems that promote and maintain the health, safety well-being, and appropriate development of children and their mothers in communities and societies to enhance the future health and welfare of society and subsequent generations [1]. Accessing essential medicines that satisfy the priority health care needs of the population is the backbone of the health care and well-being of individuals and populations [2].

The United Nations Population Fund and World Health Organization (WHO) launched the global list of priority medicines for mothers based on a global burden of disease and the evidence of efficacy and safety for preventing or treating maternal, newborn, and child mortality and morbidity [3].

The estimated global spending on health will increase from 9 to 21 trillion United States dollars in 2014 to 24 to 24 trillion United States dollars in 2040 [4]. However, lack of access to essential medicines for MCH remains a major challenge in many developing countries, where more than half of their populations lack access to essential medicines [5]. This will inevitably constrain efforts to reduce mortality and improve the health of children and mothers [6].

Nearly 4.7 million mothers, newborns, and children die each year in sub-Saharan Africa [7], 1.2 million babies die before they reach one month of age and 3.1 million children who survived their first month of life die before their fifth birthday [8]. The mortality of under-five children was 6.3 million in 2013, around 15, 11, and 7% of them were caused by pneumonia, diarrhea, and malaria, respectively, [9]. Ethiopia is one of the sub-Saharan countries with high rates of maternal and child mortality [10].

Early diagnosis and treatment with simple antibiotics could avert as many as 600,000 deaths in cases of pneumonia, whereas improving access to oral rehydration salt (ORS) would save as many of 1.3 million children die annually from diarrhea [9]. The provision of affordable, high-quality, and appropriate essential medicines is a vital component of a well-functioning health system [11] to counteract any existing barriers that might hinder medicine access [12]. Nearly 10 million lives could be saved by improved access to essential medicine [13].

In Ethiopia, pharmaceutical product availability was found to be weak, which suggests that supply chain factors may adversely affect the outcomes of MCH programs [14]. Accepting and application of MCH care policy as a general does not minimize the mortality rate of vulnerable groups due to the absence of medicines. Although few studies have been conducted, the WHO/HAI survey recommends the methodology to be applied at the state or provincial level.

## Methods

### Study area and period

A study was conducted from November 2018 to February 2019 in the public health facility of Dessie town, Ethiopia. Dessie is a town located in the Amhara region of northeast Ethiopia, 400 km away from Addis Ababa. The total population of the town is estimated to be 151,094 among this, 78,203 are females [15]. In Dessie town, there are 8 public health centers, one referral hospital, and district hospital serving for Dessie town and the surrounding nearly 8 million people.

### Study design

A cross-sectional study was conducted in public health facilities in Dessie town. Data were retrospectively abstracted from the bin card, stock card, and health commodity management information system.

### Selection of healthcare facilities

The drug outlets were selected according to the WHO/Health Action International (HAI) methodology, which has been validated to select a representative sample [9]. All public health facilities which are found in Dessie town were included in this study.

### Selection of medicines

All medicine surveys in this study were taken from the list of “priority life-saving medicines for women and children” developed by the WHO [16]. The WHO had chosen the medicines according to the global burden of the diseases and the evidence of efficacy and safety for preventing or treating major causes of maternal and child mortality and morbidity. Only registered global or regional core medicine was included in the survey. If a medicine was registered, but the stated dosage form or strength differs from that on the global/regional core list, removed the core medicine from the list and alternate form and/or strength to the supplementary list of medicines was added. Moreover, when a therapeutically equivalent medicine was widely used in addition to or instead of a medicine on the global or regional core list, the medicine was added to the supplementary list.

The recommended priority life-saving medicines for mothers are oxytocin, sodium chloride, ringer lactate injectable, and misoprostol tablets for post-partum hemorrhage; magnesium sulfate Calcium gluconate injection, hydralazine, and methyldopa tablets for severe pre-eclampsia and eclampsia; ampicillin, gentamycin, and metronidazole injectables for maternal sepsis; mispristol+mifepristone tablets for provision of safe abortion services and/or the management of incomplete abortion and miscarriage; azithromycin, cefixime capsule, and benzathine benzylpenicillin injectable for sexually transmitted infections; nifedipine capsule, dexamethasone and betamethasone injectables for management of preterm labor.

The recommended priority life-saving medicines for children under five are artemisinin combination therapy, rectal artesunate and artesunate injectable for malaria; zinc sulphate dispersible tablets and ORS sachets for diarrhea; amoxicillin (capsule), ampicillin, ceftriaxone, and gentamycin (powder for injections) for treatment of pneumonia; morphine injectable, oral liquid and granules, paracetamol, and procaine benzylpenicillin (powder for injections) for neonatal sepsis.

### Data collection tools and procedures

Data were collected by trained druggist using a standard checklist adapted from the Logistics Indicator Assessment Tool (LIAT) and WHO/HAI second edition [10]. The availability of the priority medicines in their WHO-recommended strengths and dosage formulations was assessed through physical identification in the stores and dispensaries. The pharmacy technicians were also asked to choose the reasons for stock out of medicines. The principal investigators coordinated the data collection process.

### Data processing and analysis

Data were edited and analyzed using Microsoft Excel 2010. Medicine availability was calculated as percent availability of individual medicines, mean average percent, availability across a group of medicines, and variations between sectors. Gelders S et al. (2006) described the availability of medicines in public healthcare facilities and the ranges were: < 30%, 30–49%, 50–80%, and > 80% for very low, low, fairly high, and high availability, respectively, [17].

Price and availability results were analyzed for individual medicines. Point availability was determined by the number of medicines available at time of data collection divided by the total number of medicines surveyed multiplied by hundred. Period availability was calculated by dividing the number of days the medicine available by the review period multiplied by hundred. Median Price Ratio (MPR), the ratios relative to a standard set of international reference prices calculated using the median local unit price divided by the international reference unit price. The ratio is thus an expression of how much greater or less the local medicine price than the international reference price [10]. The local currency value at the day of data collection was converted to Dollar. The medicine prices were obtained from the medical price guide issued by management science for health [18]. The ideal value for MPR was used to represent acceptable local price ratios developed by Gelders S et al. for retail patient prices in the public sector (MPR ≤1.5) [17].

The affordability of treating key health problems using standardized treatment regimens was calculated using the median prices collected during the survey. The treatment cost for an episode of illness is compared to the daily wage of the lowest-paid unskilled government worker to determine the number of days’ wages needed to pay for the cost of treatment [10]. This was done by first calculating the daily wage of the workers at the time of data collection. The formula used to calculate affordability is the total cost of medicine times thirty divided by the smallest salary unskilled government worker [19].

The total costs of medicine for the complete duration of treatments for each disease were determined and converted to daily wages. According to Robertson J (2009) criteria, medicines that cost less than a day wage were considered affordable, and those medicines with a cost greater than or equal to a day wage were considered unaffordable [20].

### Operational definitions

- **Availability:** The percentage of medicine outlets where a particular medicine was found on the day of the survey (point availability) and the last 6 months (period availability).
- **Stock out:** The frequency and duration of usable stocks unavailability in the store or a balance of zero on the stock records.
- **Affordability:** It is priced reasonably and the ability to lowest-paid unskilled government workers to pay for the cost of treatment.
- **Median price ratio:** Ratio of median retail price with the international price set by management science for health.

## Results

Ten public health facilities (2 hospitals and 8 health centers) were included in this study. In all health facilities, the responsible person for managing priority medicines was a pharmacy technician and 9 (90%) did not receive training in logistics. Averages mean years working experience store man position and at that, public health facility was 0.96 and 1.6 years, respectively.

### Availability of MCH priority medicines

**Fig. 1:**
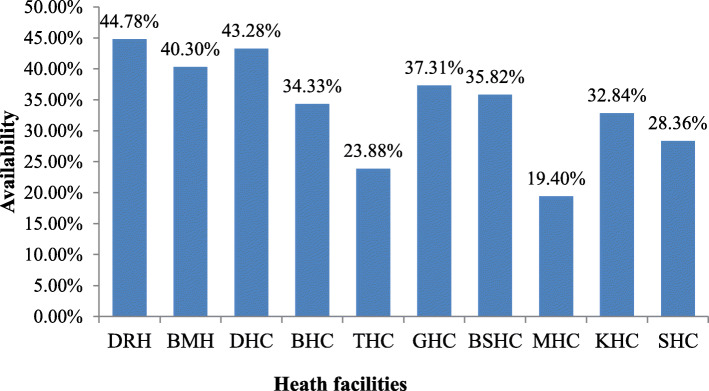
Availability of WHO priority maternal and child medicines in public health facilities, Dessie, Ethiopia

In this study, the overall mean availability of WHO prioritizing MCH medicines in the past 6 months was 34.02% (Fig. 1). From the overall medicine, 22 medicines were not found at all in public health facilities. These were ampicillin injection 250 mg, ORS sachets of 200 ml appropriate flavor, artemisinin combination therapy, artesunate rectal 50–200 mg, procaine benzylpenicillin injection 1 g, morphine with different strength, paracetamol flexible oral solid dosage forms, misoprostol 200 μg, azithromycin oral liquid 200 mg/5 mL, cefixime 400 mg, benzathine benzylpenicillin injection 900 mg, benzathine benzylpenicillin injection 1.44 g, betamethasone injection 6 mg/mL, medroxyprogesterone acetate, and artemsinin combination therapy.

The individual prioritizes medicine period availability in the past 6 months ranges from 10 to 100%. Pediatric vaccines 100% and amoxicillin dispersible scored tablets 500 mg and azithromycin 500 mg were available at 100 and 10%, respectively, (Table 1).

**Table 1:** Medicines available at public health facilities in the past 6 month, Dessie, Ethiopia

No	List of MCH medicines	Availability
1	Amoxicillin: dispersible, scored tablets 250 mg	90%
2	Amoxicillin: dispersible, scored tablets 500 mg	10%
3	Ampicillin: powder for injection 500 mg	40%
4	Ceftriaxone: powder for injection 1 g	100%
5	Ceftriaxone: powder for injection 500 mg	60%
6	Ceftriaxone: powder for injection 250 mg	10%
7	Gentamicin: injection 40 mg/ml(20 mg/mL)	90%
8	Oxygen: medicinal gas	20%
9	ORS sachets of 500 ml and 1 l, appropriate flavor	10%
10	ORS sachets of 1 l, appropriate flavor	80%
11	Zinc: 20 mg scored dispersible tablet	60%
12	Artesunate: injection dosage forms 50–200 mg	10%
13	Lamivudine + nevirapine + zidovudine — tablet 30 mg + 50 mg + 60 mg;	40%
14	Vitamin A: capsule 100,000 IU strength	50%
15	Vitamin A: capsule 200,000 IU Strength	10%
16	Morphine: granules injection 10 mg/mL	10%
17	Oxytocin: injection 10 IU in 1?ml ampoule	90%
18	Sodium chloride: injectable solution 0.9% isotonic	80%
19	Sodium lactate compound: injectable (Ringer’s lactate)	80%
20	Magnesium sulfate: injection 500 mg/ml in 10 mL Ampoule	50%
21	Calcium gluconate injection: 100 mg/ml in 10 mL ampoule	40%
22	Hydralazine: powder for injection 20 mg	50%
23	Methyldopa: tablet 250 mg	30%
24	Metronidazole: injection 500 mg	20%
25	Mifepristone + misoprostol: tablet 200 mg + tablet 200 micrograms	10%
26	Azithromycin: capsule 250 mg	10%
27	Azithromycin: capsule 500 mg	10%
28	Nifedipine: immediate release capsule 10 mg	20%
29	Dexamethasone: injection 4 mg	30%
30	Tetanus vaccine	60%
31	Oral contraceptives (pack of 2)	70%
32	Intrauterine devices and barrier methods of contraception(e.g. condoms)	40%
33	Implantable contraceptives estradiol cypionate + medroxyprogesterone acetate	60%
34	Efavirenz 600 mg + lamivudine300mg + tenofovir 300 mg	70%
35	Artemether – Lummefantrine	20%
36	Artesunate: injection dosage forms 50–200 mg	20%
37	Chloroquine 250 mg	10%
38	Oral polio vaccine	100%
39	Bacille Calmette-Guérin	100%
40	Pneumococcal conjugate vaccine	100%
41	Measles vaccine	100%
42	Tetanus Toxoid Vaccine	100%
43	Penta Valent	100%
44	Intractable Polio vaccine	100%
45	Rotavirus vaccine	100%

**Fig. 2:**
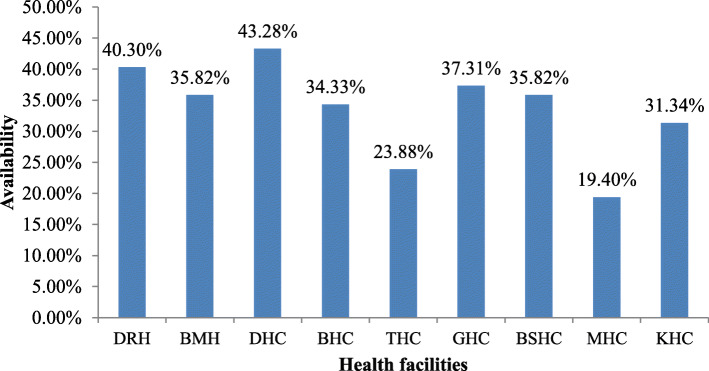
Point availability of MCH medicines in public health facilities, Dessie, Ethiopia

The mean point availability of WHO prioritizes MCH medicines in the overall health facility was 33.5% (range from 19.4 to 40.3%) (Fig. 2). On the day of the assessment, 7 medicines were stock-outs in the overall public facility, these are ampicillin 500 mg injection, ceftriaxone 1 g injection, ceftriaxone 500 mg injection, sodium chloride injectable solution, methyldopa 250 mg tablet, female condoms, and ringer lactate solution.

**Fig. 3:**
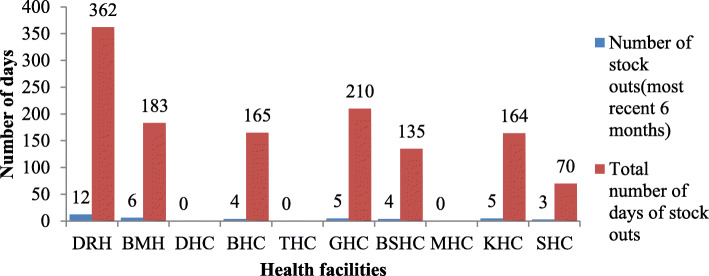
Average number and days of stock-out in public health facilities, Dessie, Ethiopia

The overall public health facilities’ average mean the number of stock-outs was 3.9, and the number of stock-out days was 128.9 in the 6 months (Fig. 3).

The stock out medicines at the period during data collection were ampicillin injection 500 mg, ceftriaxone 500 mg injection, ceftriaxone 1 g injection, and barrier methods of contraception (e.g., condoms) were not available in public health facilities (Table 2).

**Table 2:** Average number and stock out days for MCH priority medicines, Dessie, Ethiopia

S.N	list of MCH medicines	Number of stock outs (most recent 6 months)	Total number of days of stock outs
1	Amoxicillin: dispersible, scored tablets 250 mg	1	30
2	Ampicillin: powder for injection 500 mg	5	165
3	Ceftriaxone: powder for injection 1 g	7	204
4	Ceftriaxone: powder for injection 500 mg	3	195
5	Gentamicin: injection 40 mg/ml(20 mg/mL)	1	10
6	Morphine: granules injection 10 mg/mL	1	15
7	Oxytocin: injection 10 IU in 1-ml ampoule	1	25
8	Sodium chloride: injectable solution 0.9% isotonic	4	95
9	Sodium lactate compound: injectable (Ringer’s lactate)	4	58
10	Magnesium sulfate: injection 500 mg/ml in 10-ml mL Ampoule	1	30
11	Calcium gluconate injection: 100 mg/ml in 10-ml mL ampoule	1	60
12	Hydralazine: powder for injection 20 mg	1	15
13	Methyldopa: tablet 250 mg	3	90
14	Dexamethasone: injection 4 mg	2	140
15	Tetanus vaccine	1	7
16	Oral contraceptives (pack of 2)	1	60
17	Intrauterine devices and barrier methods (e.g. condoms)	2	90

### Affordability of MCH medicines

The wages required to purchase the standard treatment for severe pneumonia with ceftriaxone 1 g injection and post-partum hemorrhage with oxytocin was 8.01 and 0.38 days wages to pay for the treatment required for unskilled government worker income (960) birr, respectively, (Table 3).

**Table 3:** Affordability of WHO prioritize MCH medicines in public health facilities, Dessie, Ethiopia

No	Condition	List of MCH medicines	Treatment schedule	Days wages to pay for treatment
1	Pneumonia	Amoxicillin: dispersible, scored tablets 250 mg	25 mg/kg ^a^14.5 kg PO BID for 7 days =21 cap	0.38
2	severe Pneumonia	Ceftriaxone: powder for injection 1 g	80 mg/kg^a^14.5 kg IV daily for 10 days = 12 vial	6.58
3	severe Pneumonia	Ceftriaxone: powder for injection 500 mg	80 mg/kg^a^14.5 kg IV daily for 10 days = 24 vial	8.01
4	Neonatal sepsis	Gentamicin: injection 40 mg/mL(20 mg/ml)	5 mg/kg^a^14.5 kg IV daily for 10 days = 10 amp	1.17
5	Diarrhea	ORS sachets of 1 l, appropriate flavor	75 ml/kg^a^ 14.5 kg = 2 sachet	0.32
6	Postpartum hemorrhage	Oxytocin: injection 10 IU in 1- mL ampoule	10 units IM stat	0.25
7	Postpartum hemorrhage	Sodium chloride: injectable solution 0.9% isotonic	1000 ml	1.08
8	Postpartum hemorrhage	Sodium lactate compound: injectable (Ringer’s lactate)	1000 ml	1.22
9	Severe pre-eclampsia and eclampsia	Hydralazine: powder for injection 20 mg	20 mg/ml IM BID daily	5.02
10	Prevention of tetanus	Tetanus antitoxin	10,000 IU IM after skin test	3.45

^a^Average weight under five years old in Ethiopia is 14.5 kg [21, 22]

### Price of MCH medicines

**Fig. 4:**
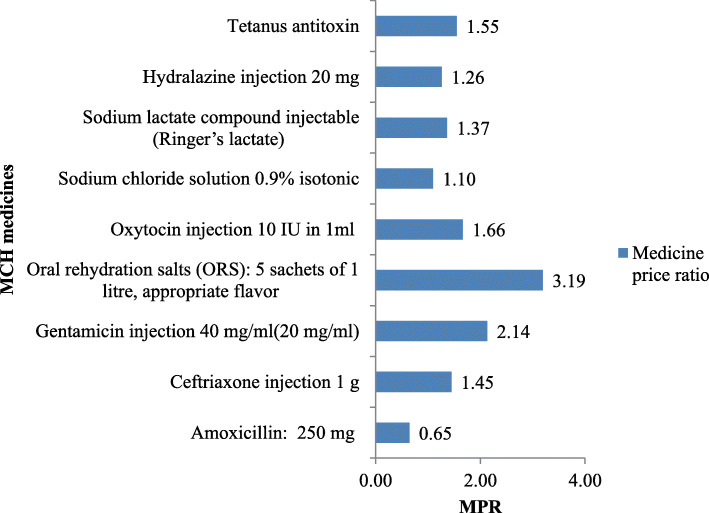
MPR the WHO prioritizes MCH medicines in public health facilities, Dessie, Ethiopia

The median price ratio of priority lifesaving MCH medicines in public health facilities ranged from 0.65 to 3.19. For amoxicillin 250 mg dispersible scored tablets and ORS sachets of 1-l appropriate flavor were 0.65 and 3.19, respectively, (Fig. 4).

Amoxicillin dispersible 250 mg 1(10%), calcium gluconate injection 100 mg 2(20%), gentamicin injection 40 mg/ml (20 mg/mL) 5(50%), implantable contraceptives estradiol cypionate + medroxyprogesterone acetate 2(20%), depot injection 150 mg/mL in 1-mL vial 1(10%), hydralazine injection 20 mg 2(20%), magnesium sulfate injection 500 mg/mL 1(10%), and methyldopa tablet 250 mg 1(10%) was found a surplus of before resupply in public health facility.

Health professionals working in public health facilities pinpointed the reason for stock-outs of priority lifesaving MCH medicines and pharmaceutical fund supply agencies did not supply adequate products 9 (90%), wastage due to the expiry of products 6 (60%) and lack of information about MCH medicines 3 (30%) were the stated reasons.

## Discussion

Universal health coverage is a comprehensive health system approach that facilitates a wide range of health services and significantly improves the life expectancy of patients [23]. Medicines are an essential component of healthcare delivery in any country. In developing countries, equitable access to safe and affordable medicines is crucial to the health and well-being of people. Despite progress made so far in the areas of public health, medicines remain the single most vital factor in the maintenance of health and the treatment of diseases [24].

In this study, the overall mean period availability of the WHO prioritized MCH medicines in public health facilities in the past 6 months was 34.02%. These findings were similar to the study conducted by Abrha (2018), where the availability of priority life-saving was 34.1% [25]. This is also consistent with a study conducted by Sautenkova N et al. (2012) [26].

According to Gelders S et al. (2006) criteria, [17] the present finding was very low. Lower periodic availability was also reported as compared to a study done by Prinja S et al. (2015) in India, where the overall mean availability of medicines was 45.2 and 51.1% Punjab and Haryana, respectively, [27]. In the Republic of Moldova, the mean availability in the public sector was 51.2% [28] and 46% mean availability was reported in Guatemala by Anson et al. (2012) [29].

The variations in the availability of WHO priority MCH medicines among studies might be due to poor inventory management systems and inadequate allocation of funds to health facilities to purchase sufficient amounts of MCH medicines. Financial constraints or inefficient budget utilization for the treatment of chronic and acute conditions and the absence of dollar currency to purchase vital medicines from outside countries have more worsen problems in medicine availability in the health sector.

The government also does not allocate sufficient funds to mobilize MCH care services and neglect such services to non-governmental organizations to facilitate the program, which creates a question on a mandate for the unavailability of medicines. Although priority lifesaving drugs are used for the treatment of various diseases conditions in children and adults [30], many deaths were due to conditions that could be prevented or treated with access to vital medicines at public health facilities [31]. Health insurance coverage and the package of services covered by health insurance plans were shown to increase the affordability of a vast portion of the medical goods and services that are commonly paid for out-of-pocket expenditure [32].

Medicines such as cefixime 400 mg, betamethasone injection 6 mg/mL, medroxyprogesterone acetate, and artemsinin completely absent in public health facilities. This was similar to the absence of essential medicines in the northern part of Ethiopia [25]. This might be due to the longer time required to update newly emerging WHO priority medicines to treatment guidelines at the country level and letter in the hospital and health center level. The absence of a legal accusation system for the non-availability of WHO priority MCH medicines is also the claimed reason.

In this study, the overall public health facility average mean number of stock-out days was 128.9 over 6 months. A high number of stock-out days as compared to the study conducted by Fentie (2015) in Gondar (30.5 days) [33] and lower than Kibira (2017) study 13 reproductive, maternal, newborn, and child health commodities and stock-outs ranged from 14 days [34]. This difference might be attributed to poor stock management, quantification, and procurement practices.

The mean average number of stock out was 2.29, and once for individual drugs like oxytocin injection and amoxicillin dispersible tablets 250 mg which is lower (9.1) compared to the stock-outs of essential health products in Mozambique and for drugs such as oxytocin, the number of stock out was 2.6 at the district level [35]. This finding also lower than that done by Getahun et al. (2015), where amoxicillin 250 mg scored dispersible tablets number of a stock-out was 33 [36]. This might be due to differences in the study period; they conducted a longer period (almost 3 years). Regular and consistent availability of the necessary medicines is the topmost priority for any health sector. A shortage of pharmaceuticals adversely affects the quality of health care and the condition will be severe if stock out is prolonged.

Inadequate supply from the supplier, lack of information about MCH medicines and expiry were the mentioned reasons for stock out of WHO-prioritized MCH medicines. This reason was similar to the study conducted by Getahun et al. [36]. This might be because the pharmaceutical supply to public health facilities throughout the country is being managed by the same supplier [37] as a result of the lack of a strong information communication system between the supplier.

The mean average point availability in the overall health facility was 33.5%, and on the day of the assessment, stock-outs of medicines were ampicillin 500 mg injection, ceftriaxone 1 g injection, ceftriaxone 500 mg injection sodium chloride injectable solution, methyldopa 250 mg tablet, female condoms, and ringer lactate solution. This finding was lower than that of a study in Malawi reported that the overall mean availability was 60% and stock-outs of at least one product on the day of the assessment [14]. This discrepancy was attributed to the expiry of medicines before use.

In the present study, the lowest-paid government worker in Dessie town unable to purchase the product to cover the full course of treatment like ceftriaxone 1 g injection, hydralazine 20 mg injection, and tetanus antitoxin, which require more than daily wages. The results were lower than studies in China, where the affordability of amoxicillin 250 mg for the treatment of severe pneumonia was 1.4 [38]. A study conducted by van Mourik M revealed that for treatment of infectious disease with ceftriaxone 1 g injection, 15 days wages required [39]. The lowest-paid government worker was unable to afford due to their lower financial income [20]. The rising out-of-pocket health spending continues to threaten the affordability of medical care [40, 41].

The MPR was 4 (55.5%) and was considered to be high. The ORS sachets of 1 l were 3.19 times greater, gentamycin injection was 2.14 times greater, and Amoxicillin 250 mg 0.65 times lower than international median prices. This was a higher MPR (1.53) as compared to the Sado E and Sufa A study [42]. Wang X et al. (2014) study revealed that the MPR for ORS was (2.22) and the MPR for amoxicillin (1.82) [43]. Such differences among studies might be due to price inflation of the dollar exchange rate and profit markup range added to retail patient prices for medicines. Some medicines supplied from donors without a price and this medicine, price setting are used for auditing purposes. Besides, high prices were more pronounced when purchasing done from private wholesales during stock out. Although the cost of health care services for MCH, including WHO prioritize medicines was covered by the government, the burden still relies on the government. Catastrophic household health expenditure from out-of-pocket expenditure on medicines could plunge patients into poverty [44].

The availability and affordability of medicines with reasonable prices in public health facilities have strong clinical implications for reducing maternal, newborn, child morbidity, and mortality. This study has significant for the government, stakeholders, managers, and policymakers to develop national regulations and strategies to enhance access to WHO priority MCH medicines for public health facilities.

The point and period availability formula consider the availability of medicines ranging from low to overstock regardless of health institutions need. Nevertheless, in the real world, the availed WHO prioritize MCH medicines might not be sufficient and do not show how much amount of medicines are adequate. The affordability formula failed to incorporate the wages of people in the informal sector who were below the salary income of the government. Moreover, in the MPR calculation, the international price used for comparison was the list of medicine by management science for the health 2015 version and does not update yet, and it has limitations in revealing the MPR ratio at present time. The WHO/HAI price project recommended collecting the price on targeted medicines at state or provincial the result was specific to the surveyed province and thus cannot be generalized to the country.

## Conclusion

The average mean availability of WHO prioritized MCH medicines in the past 6 months and point mean availability was very low and a high number of stock-outs. One-third of WHO prioritized medicines were completely unmanaged in all health facilities. From the WHO priority list of medicines, some of the products were unaffordable. Improving availability and strict inventory control are recommended to have a steady supply of these essential medicines to improve the quality of health services.

## Data Availability

All data generated or analyzed during this study are included in this published article.

## Abbreviations

HAI: Health Action International
MCH: Maternal and child health
MPR: Median Price Ratio
ORS: Oral rehydration salt
WHO: World Health Organization
